# Sustainable agriculture in the era of omics: knowledge-driven crop breeding

**DOI:** 10.1186/s13059-020-02073-5

**Published:** 2020-06-26

**Authors:** Qing Li, Jianbing Yan

**Affiliations:** National Key Laboratory of Crop Genetic Improvement, Huazhong Agricultural University, Wuhan, 430070 China

## Challenges in food production

Global population has reached up to 7.8 billion and is expected to exceed 10 billion by 2055 (https://countrymeters.info/cn/World). Such rapid population increase presents a great challenge for food supply. On the one hand, more grains are needed to provide basic calories for humans. On the other hand, the rising living standard leads to a changing diet habit towards higher average consumption of livestock and dairy products, especially in developing countries. Thus, crop yield boost is needed to fill the gap between food production and demand. Meanwhile, food nutritional values are of more interest to accommodate industrialized modern lives.

The instability of food production caused by global climate change is another great challenge. Since 1880, the earth’s temperature has risen by more than one degree (https://earthobservatory.nasa.gov/world-of-change/global-temperatures), and the warming rate is becoming more rapid in recent decades, with more frequent extreme climate change including high temperature, drought, and floods. This requires future crops to adapt to this new and unpredictable environment. Crop varieties resistant to biotic stresses are also needed as plant disease and insects are expected to be impacted by climate change.

More importantly, we need a food production system that can simultaneously satisfy societal demands and long-term development. Since the Green Revolution in the 1960s, farming is heavily dependent on high input of nitrogen and pesticides. This leads to environmental pollution which is not sustainable in the long run.

Therefore, a new breeding scheme is urgently needed to enable sustainable agriculture; including new strategies to develop varieties and crops that have high yield potential, high yield stability, and superior grain quality and nutrition; nevertheless, less consumption of water, fertilizer, and chemicals should also be considered for environmental protection purposes.

## Opportunities from omics knowledge and new emerging technologies

**Fig. 1:**
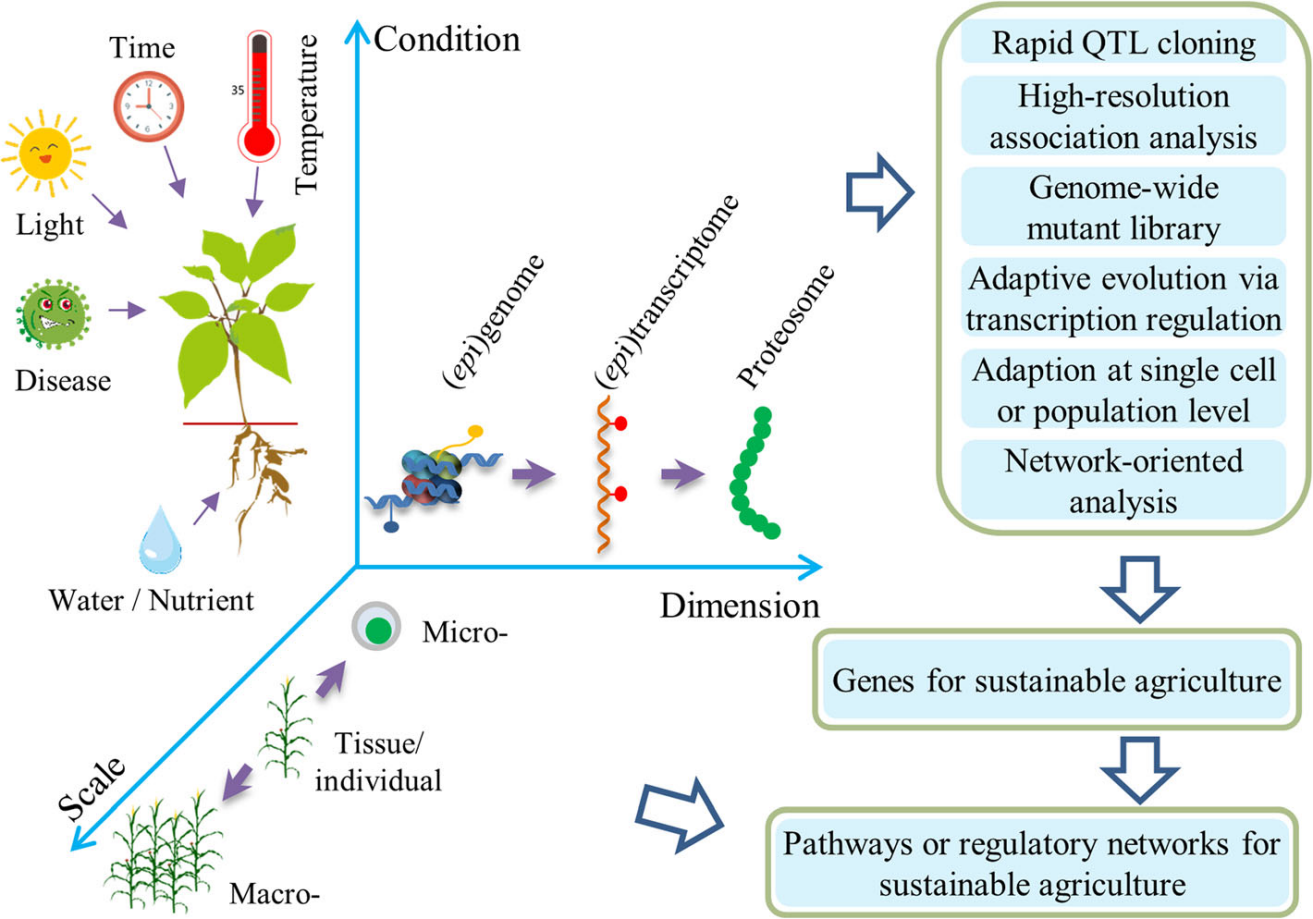
Omics-driven characterization of genes and pathways important for sustainable agriculture

While we are facing challenges, there are also great opportunities, especially with flourishing developments in omics technologies. High-quality reference genomes are becoming available for thousands of species, with some species having more than one reference genome. The genome-wide re-sequencing of diverse varieties enables the identification of core- and pan-genomes. This expands our understanding on crop domestication and improvement. Omics data are being generated at multiple layers, including but not limited to genomes, epigenomes, transcriptomes, epitranscriptomes, and proteomes (Fig. 1). Many of the previous omics data were generated from tissues of a single genotype and is now extending to micro- and macro-scales [1, 2]. Omics data is also being generated under different environmental conditions [3]. Currently, it is feasible to generate omics data for any species at a reasonable cost. It is important to note that many matching informatics tools are rapidly developed to mine biological meanings from the omics data.

One application of omics data is to identify functional genes that are relevant for sustainable agriculture (Fig. 1). These genes can then be manipulated to develop new varieties and crops for sustainable farming. Specifically, successful identification of genes using omics data is manifested in the following aspects.

First, omics knowledge accelerates QTL (quantitative trait loci) cloning. A recent study showed that functional genes underlying QTL can be successfully identified within 1–2 years (compared to > 3 years before) when genomic big data is coupled with careful population design [4]. Second, omics-based association analysis increases mapping resolution to gene level. Re-sequencing of a panel of diverse lines identifies a vast pool of genetic variations, especially structure variations which are more likely to have a phenotypic effect than SNPs [5]. Third, omics enables the rapid identification of important genes with limited natural variations. Genome-wide mutation libraries, in combination with genome-wide or targeted sequencing, allow the dissection of key genes that have not been utilized in current breeding programs [6]. Fourth, comparative transcriptomic analyses under different environmental conditions allow the identification of genes that are important for adaptation [3]. Fifth, omics at single-cell scale identify cell types critical for environmental adaptation [1]. Finally, an integration of multi-omics data to develop a system network is expected. This will enable a network-oriented identification and prioritization of key genes and pathways.

## Precision breeding enables sustainable agriculture

**Fig. 2:**
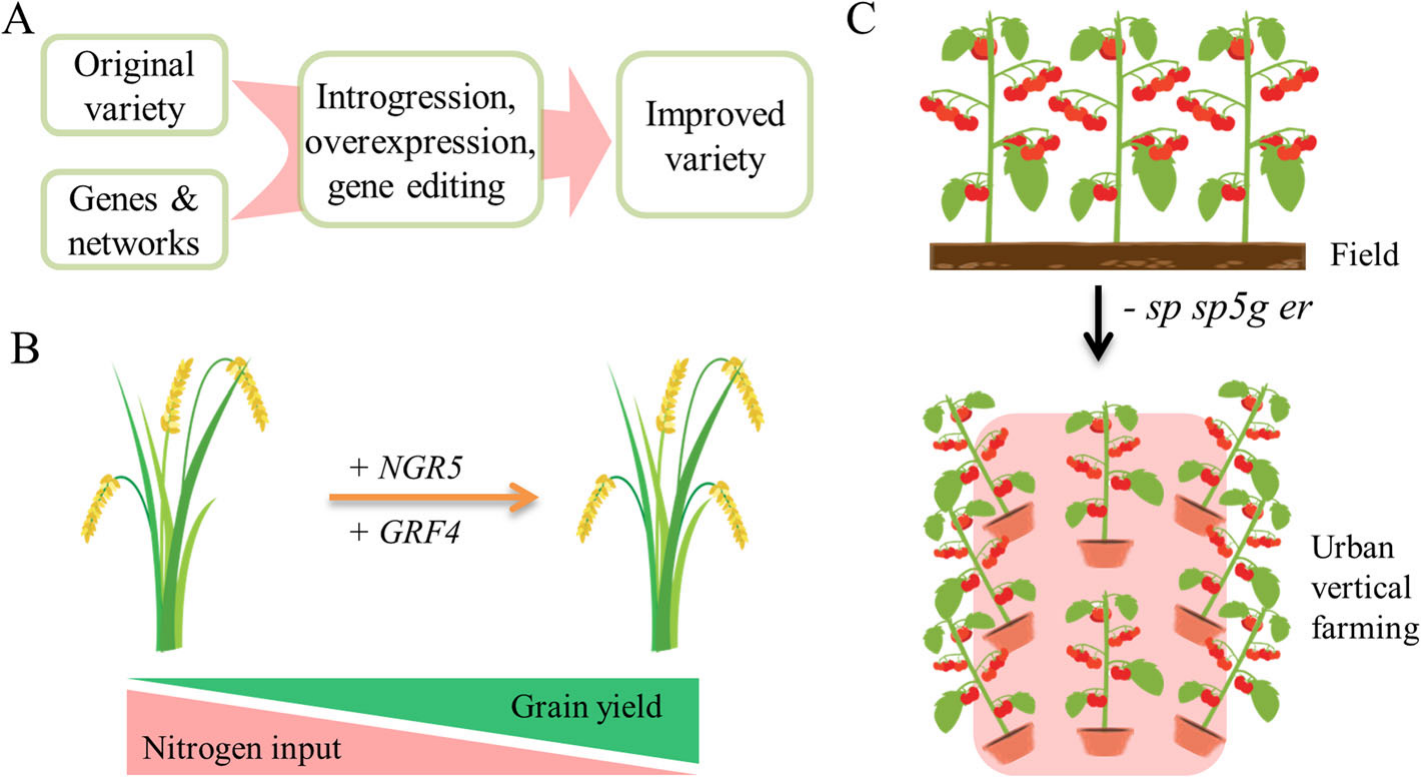
Breeding crops for sustainable agriculture. **a** Strategies to improve current varieties with the knowledge gained through omics data. **b** An example to show increase in gain yield and decrease in nitrogen input when two genes (*NGR5* and *GRF4*) were introduced into the original variety [7, 8]. *GRF4*, *growth-regulating factor 4*; *NGR5*, *nitrogen-mediated tiller growth response 5*. **c** An example to illustrate changes in plant architecture which makes it suitable to cultivate under urban environment by knocking out three genes [10]. *sp*, *self pruning*; *sp5g*, paralog of *sp*; *er*, *erecta*

The knowledge gained from omics data, in combination with new technologies like targeted gene editing, can help to breed new varieties and crops for sustainable agriculture (Fig. 2a). The introgression/over-expression of genes responsible for high nitrogen use efficiency (NUE) into the green revolution variety represents a nice feasible route for developing varieties for sustainable agriculture [7, 8]. The Green Revolution during the 1960s was enabled by the adoption of semi-dwarf varieties, which can resist lodging but have reduced NUE [7]. The high-yield potential is achieved by intense nitrogen fertilization, which degrades the environment. The identification of two functional genes underlying NUE provides a possibility to combine the two beneficial traits, semi-dwarfism and high NUE. Indeed, integration of these two NUE genes into green revolution varieties enables high yield with reduced nitrogen application [8]. Both genes have naturally occurring favorable alleles and can be introgressed into other varieties. Besides, both genes increased NUE when expression levels were increased. Therefore, it is possible to over-express them, or manipulate the promoter regions of both genes using CRISPR/Cas9 technology to boost their expression in the elite semi-dwarf varieties, and achieve high-yield in an environmentally friendly manner (Fig. 2a, b). This is an elegant example to show that a key gene can not only ensure high yield potential, but also reduce the input of nitrogen fertilizer to promote sustainable development. Gene pleiotropy is a common biological phenomenon, genes with multiple effects may play different roles through different pathways which can be precisely regulated to meet different needs and achieve the goal of precise breeding by using genome editing technologies.

## Re-domestication and omics-driven de novo design of new crops

With global climate change, many plants, especially those at mid-to-high latitudes are at high survival risk. Their inability to deal with the ongoing climate change can not only affect the survival of themselves but also the animals and people that depend on them. Therefore, climate-resistant varieties and crops with high and stable productivity need to be developed. So far, crop genetic improvement relies on artificial selection of existing natural variations at low efficiency. Nevertheless, the genetic bottleneck during domestication resulted in a loss of a vast amount of genetic variations, which may include the favorable variations that enable the plants to adapt to different environmental conditions. It is not a surprise that traditional crop genetic improvement cannot adapt to current high demand of yield and environment sustainability. Therefore, we have to re-visit the wild ancestors of current crops to harness the favorable variations. Re-domestication of wild ancestors is possible with the knowledge of domestication and the new technologies [9]. During the re-domestication process, we can purposely incorporate beneficial genes to develop new varieties with environment adaptation advantages and high yield potential at the same time. Alternatively, novel variations can be created using technologies like CRISPR/Cas9. This is particularly intriguing for genes that may not have the best favorable allele in nature.

In addition to developing climate-resistant varieties and crops, growing crops in well-controlled urban environments is another approach that can support sustainable agriculture [10]. For example, the vertical farming system cultivates plants under restrictive growth conditions, and thus, optimizes land use (Fig. 2c). Though per plant yield is low, high productivity is achieved through high planting-density and rapid crop cycling. This is a new but sustainable idea for agriculture development, which has rarely been practiced in the past breeding process because of the lack of compact and rapid cycling variety that can be cultivated under restrictive spaces since our current varieties and crops are usually bred to achieve maximum productivity under typical field conditions.

## Perspective

We are in an era with enormous omics data. An integration of omics data will enable a rapid and high-throughput identification of many genes simultaneously for a relevant trait. This will change our current research paradigm fundamentally from single gene analysis to pathway or network analysis. With the enormous knowledge of domestication and improvement gained from omics data, in combination with the new gene editing technologies, we can create future crops via a three-step road map [9]. In this way, our future crop breeding will not only satisfy diverse human needs, but also adapt to the revolution in the farming system. The knowledge gained through omics data will ensure such a success, contributing to the development of sustainable agriculture.
